# Hsp90 and Its Co-Chaperones in Neurodegenerative Diseases

**DOI:** 10.3390/ijms20204976

**Published:** 2019-10-09

**Authors:** Anastasiia Bohush, Paweł Bieganowski, Anna Filipek

**Affiliations:** 1 Nencki Institute of Experimental Biology, Polish Academy of Sciences, 3 Pasteur Street, 02-093 Warsaw, Poland; a.bohush@nencki.gov.pl; 2 Mossakowski Medical Research Centre, Polish Academy of Sciences, 5 Pawińskiego Street, 02-106 Warsaw, Poland; pawelb314@gmail.com

**Keywords:** Hsp90, co-chaperones, Alzheimer’s disease, Parkinson’s disease, Huntington’s disease, prionopathy, Hsp90 inhibitors

## Abstract

Proper folding is crucial for proteins to achieve functional activity in the cell. However, it often occurs that proteins are improperly folded (misfolded) and form aggregates, which are the main hallmark of many diseases including cancers, neurodegenerative diseases and many others. Proteins that assist other proteins in proper folding into three-dimensional structures are chaperones and co-chaperones. The key role of chaperones/co-chaperones is to prevent protein aggregation, especially under stress. An imbalance between chaperone/co-chaperone levels has been documented in neurons, and suggested to contribute to protein misfolding. An essential protein and a major regulator of protein folding in all eukaryotic cells is the heat shock protein 90 (Hsp90). The function of Hsp90 is tightly regulated by many factors, including co-chaperones. In this review we summarize results regarding the role of Hsp90 and its co-chaperones in neurodegenerative disorders such as Alzheimer’s disease (AD), Parkinson’s disease (PD), Huntington’s disease (HD), and prionopathies.

## 1. Introduction

Folding into a defined three-dimensional structure is crucial for proteins to achieve functional activity in the cell. Proteins often fail to preserve their structure when cells are exposed to stress, such as high temperature, toxic chemicals, and others. These factors often lead to protein misfolding and formation of protein aggregates [1]. Accumulation of these aggregates is a burden for the cell, since it leads to major dysregulation of cellular metabolism [2]. Protein aggregates can be found in at least 30 different human diseases, including various neurodegenerative diseases. A recent study on a mouse model of Alzheimer’s disease (AD) provides compelling evidence that the presence of insoluble tau in neuronal cells alters the solubility of hundreds of other proteins, causing an overall failure of cell homeostasis [3].

Molecular chaperones and their co-chaperones are proteins that assist other proteins in proper folding into three-dimensional structures to attain functionality. The key role of molecular chaperones is to prevent protein aggregation, especially under conditions of cellular stress. Expression of chaperones is often induced by heat shock, oxidative stress, toxic chemicals, or inflammation [4]. During aging, the imbalance between chaperone/co-chaperone levels and activity in neurons seems to be responsible for a decline in protein folding [5]. Thus, in general, one may assume that this imbalance contributes to the development of age-related neurodegenerative diseases.

Hsp90 is an essential protein in all eukaryotes, and a major regulator of protein folding in the cell [6]. Extensive research provides a bulk of evidence to support a decisive role of Hsp90 and its co-chaperones in folding and degradation of hallmark proteins involved in various neurodegenerative diseases [7]. Thus, in this review we focus on the role of Hsp90 and its selected co-chaperones in these disorders, in particular in AD, Parkinson’s disease (PD), Huntington’s disease (HD), and prionopathies. Also, we outline some beneficial in vitro effects of Hsp90 inhibitors which, in the future, might be useful in therapeutic approaches.

## 2. Hsp90—A Basic Overview

Hsp90 is highly abundant in eukaryotic cells and accounts for 1–2% of total cellular proteins. Under stress conditions the amount of this protein can increase, even up to 4–6% [8,9,10]. Its protein sequence is highly phylogenetically conserved from bacteria to humans [8]. In the cell, Hsp90 associates with various substrate proteins, collectively called clients. Hsp90 is responsible for their proper maturation, activation and degradation. Essentially, most Hsp90 clients, including tau, α-synuclein, huntingtin, kinases, transcription factors, steroid hormone receptors, and E3 ubiquitin ligases, are structurally unrelated [8,11,12,13,14]. Many of these proteins are regulators of different cellular processes, such as protein folding and degradation, cell growth, chromatin remodeling, cellular trafficking, differentiation, and others [6]. A current list of Hsp90 clients can be found at https://www.picard.ch/downloads/Hsp90interactors.pdf. In mammals, there are two major cytoplasmic isoforms of Hsp90, a stress inducible Hsp90α, and a constitutive Hsp90β. Hsp90 homologs are also present in other cellular compartments, for instance Grp94 in the endoplasmic reticulum, or Hsp75/TRAP1 in the mitochondrial matrix [15]. Structurally, Hsp90 contains three domains: the N-terminal ATP-binding domain, middle domain, and the C-terminal dimerization domain. The Hsp90 dimer undergoes conformational changes, distinct conformations being stabilized by interactions with co-chaperones and ATP. ATP hydrolysis drives the Hsp90 chaperoning cycle, and is essential for proper folding of the client proteins [16,17,18].

Hsp90 can also be regulated by other factors such as heat shock factor (HSF1), or post-translational modifications (PTMs) [19,20,21,22]. HSF1 is a major regulator of heat shock response in eukaryotes [23]. When repressed, HSF1 is associated with Hsp90 but during stress HSF1 is released, homotrimerises and binds to heat-shock-factor elements (HSE) on the promoter sequence of gene encoding Hsp90. In consequence this association leads to rapid up-regulation of Hsp90 [24]. PTMs, namely phosphorylation, acetylation, S-nitrosylation, oxidation, described and reviewed in detail elsewhere, represent another mechanism regulating Hsp90 activity [20,21,22]. PTMs lead to alterations in client/co-chaperone recognition and binding. Also, Hsp90 domain organization is subject to conformational changes due to PTMs [19,25,26]. The most studied and important is phosphorylation. This modification has been proposed to positively influence maturation of some clients, while hyperphosphorylation is negatively correlated with Hsp90 chaperone activity in vivo [19,27]. Other modifications, namely acetylation and S-nitrosylation, halt the strong interaction between Hsp90 and client proteins, sometimes leading to client destabilization and degradation [25,28,29]. Another type of Hsp90 regulation, which includes co-chaperones, relies on mediation of Hsp90 ATPase activity. Generally, co-chaperones are proteins capable of interacting with Hsp90 and assisting its function, while they do not rely on Hsp90 for their own folding and stability. Interestingly, unlike other forms of Hsp90, cytosolic Hsp90 depends on co-chaperones’ help in regulation of ATPase hydrolysis. The list of Hsp90 co-chaperones could be found at https://www.picard.ch/downloads/Hsp90interactors.pdf, while the list of co-chaperones mentioned/described in this review is placed in Table 1.

## 3. Hsp90 and its Co-Chaperones in AD

AD is a progressive neurodegenerative disorder characterized by cognitive impairment accompanied by language, visuospatial, and motor dysfunctions [30]. The histopathological hallmark of AD is the extracellular accumulation of amyloid-β (Aβ) in senile plaques and formation of intracellular neurofibrillary tangles (NFTs) [31]. Amyloid plaques consist of β-amyloid (Aβ) peptides, which are derived as a result of cleavage of the amyloid precursor protein (APP) [32]. NFTs are composed of the microtubule-associated protein, tau [33]. It was generally accepted that in neurons the physiological function of tau is to stabilize microtubules [34,35,36], until recent studies have suggested that rather than stabilizing, tau helps microtubules to achieve greater length [37]. Apparently, the physiological function of tau still remains unclear. Under pathological events in the brain, this protein becomes hyperphosphorylated and susceptible to misfolding and dissociation from microtubules. This is followed by formation of abnormal tau aggregates and their polymerization into NFTs [38]. Hyperphosphorylated tau is detected in the brain of adolescent animals, and its level increases in the fibrillary form throughout aging [39]. Another research on a mouse model of tauopathy shows that abnormal tau phosphorylation is the main cause of cognitive decline, and that Hsp90, together with its co-chaperones, is able to regulate tau phosphorylation and dephosphorylation [40,41]. Some other work demonstrates that inhibition of Hsp90 causes a decrease in phosphorylation of tau; importantly, the effect does not lead to HSF1 activation [42]. Furthermore, several lines of investigation support the idea that Hsp90 regulates tau phosphorylation status in an indirect way that is through stabilization of tau kinases [43,44]. Hyperactivation of tau kinases is thought to contribute to tau pathogenesis [45]. Accordingly, it has been suggested that Hsp90 inhibition is a promising way to reduce the activity of tau kinases [46]. In cellular and mouse models of tauopathy it was found that inhibition of Hsp90 leads to reduction in the activity of the Cdk5 tau kinase and, subsequently, in the level of tau aggregates [47]. It is well known that not only kinases but also phosphatases are important for maintaining a proper level of phosphorylated tau. For example, PP5 and CacyBP/SIP phosphatases, which also serve as Hsp90 co-chaperones, can dephosphorylate tau [48,49]. One study has suggested that reduction in PP5 activity in an AD brain may contribute to hyperphosphorylation of tau [48,50]. It cannot be excluded that increase in tau phosphorylation during aging may be due to the loss of activity of these phosphatases.

Phosphorylation of tau can also be regulated by a Hsp90 co-chaperone, Cdc37, which co-localizes and interacts with tau in the human brain. It was found that knock-down of Cdc37 in HeLa cells might influence the stability of tau kinases, such as Cdk5 and Akt. Supporting this finding, suppression of Cdc37 destabilizes tau and leads to its clearance, whereas Cdc37 overexpression maintains tau level in these cells. The level of Cdc37 significantly increases with age and it has been suggested that this change contributes to the tau phosphorylation profile, consequently altering toxicity and stability of this protein [51].

It is worth mentioning that tau may adopt many transitional conformations and each of these conformations may have a toxic potential [52]. Thus, it is essential to execute mechanisms to cleave abnormal tau in neuronal cells. One of these mechanisms involves the CHIP protein, a Hsp90 co-chaperone, which mediates degradation of abnormal/modified tau due to its E3 activity. In HeLa cells CHIP triggers ubiquitination of tau via the ubiquitin-proteasome proteolytic pathway [42,53]. Also, another co-chaperone, STI1/Hop, which helps to orchestrate the Hsp70-Hsp90 complex, plays a crucial role in the clearance of tau protein. Loss-of-function mutations in the gene encoding STI1/Hop trigger toxic tau accumulation in the fly model of tauopathy [54]. There are also some data showing that in AD the level of Sgt1, another Hsp90 co-chaperone, is lower [55]. Altogether, these results suggest that age-related loss of function/activity of some co-chaperones may be essential for maintaining brain homeostasis and for etiology of neurodegenerative diseases.

Another class of Hsp90 co-chaperones is represented by proteins that can specifically rearrange folding at proline residues. To this class belong immunophilins, such as FKBP51 or FKBP52. Notably, proline residues, which have a high potential for aggregation, are abundant among intrinsically disordered proteins; for example, tau has 40 such residues [11]. One study identifies FKBP51, unlike other co-chaperones, to be significantly up-regulated in aged and AD brains [56]. Some other data indicate that FKBP51 is involved in tau stabilization [57,58].

In contrast to FKBP51, the level of FKBP52 is abnormally low in AD brains [59]. When the gene expressing FKBP51 was knocked-down in HeLa cells, a dramatic decrease in total tau level, compared to control or FKBP52 siRNAs, was observed. FKBP51 overexpression caused an increase in phosphorylated tau and total tau level (by 80%) in cells cultured in vitro, while FKBP52 overexpression had no effect. It has also been speculated that FKBP51 might maintain tau by impairing its ubiquitination. Further support for this idea is provided by the observation that in cells with stable tau and FKBP51 overexpression, tau ubiquitination is decreased [57]. FKBP52 is detected together with tau in the autophagy-endolysosomal system in some AD neurons, and a decrease in FKBP52 correlates with NFT formation. Additional experiments utilizing tau-inducible neuroblastoma SH-SY5Y cells demonstrate a release of FKBP52 from the cell under an inhibition of autophagy. These findings explain the correlation between early neuronal accumulation of the pathological form of tau, and abnormal release of FKBP52 from NFT negative neurons in AD brains [59]. This suggests that FKBPs may be promising drug targets in AD therapy; the drug design, however, will certainly have to overcome challenges presented by FKBPs structural homology. Nonetheless, specific FKBPs can be targeted, owing to a substantial diversity in the conformational flexibility of protein domains [60]. Several studies demonstrate that acute treatment with an inhibitor of immunophilins restores memory function in cognitively impaired animals. This work has been performed on a well-established model of AD and on wild type mice acutely injected with Aβ oligomers [61,62,63]. Another study from the same research group demonstrates significant reduction in the incidence of AD in patients treated with FK506 [64]. Similarly to the effect observed for FKBP52, depletion of another Hsp90 co-chaperone, p23, caused a significant decrease in total and phosphorylated tau level in human cells [42].

Microglia plays a central role in the pro-inflammatory response in AD. In order to understand Hsp90 contribution to immune response in AD, researchers studied the effect of exogenous recombinant Hsp90 on isolated microglial cultures from rat and mouse brains. It was found that Hsp90 increased phagocytosis in microglia and, subsequently, promoted the clearance of Aβ. This effect is achieved through induction of IL-6 and TNFα production and activation of TLR4 [65]. Notably, Hsp90 seems to have a dual effect. Activation of microglia leads to lower Aβ accumulation due to an increase in Aβ phagocytosis, clearance, and degradation, which prevents the formation of amyloid plaques in the brain. Chronic microglial activation, which is a hallmark of AD, leads to the release of pro-inflammatory cytokines, which initiate a pro-inflammatory cascade and contribute to neuronal damage [66].

There are several studies in vitro on the influence of Hsp90 and its co-chaperones on tau aggregation. Aha1, a co-chaperone which stimulates Hsp90 ATPase activity, enhances tau aggregation in vitro and increases accumulation of oligomeric and insoluble tau in a mouse model of this disease. Moreover, inhibition of Aha1 reduced tau accumulation in cultured cells. Also, overexpression of Aha1 in a mouse model caused neuronal loss and cognitive impairments [11]. Interestingly, another in vitro study suggests a role of Hsp90 in blocking Aβ aggregation at early stages. Monomeric Aβ and its oligomers were more susceptible to Hsp90 influence, while fibrils were less affected [67].

## 4. Hsp90 and its Co-Chaperones in PD

PD is the second most common neurodegenerative disorder after AD. It is characterized by a progressive loss of dopaminergic neurons in substantia nigra, and accumulation of toxic α-synuclein aggregates in specific inclusions called Lewy bodies (LB). Clinical signs of PD are progressive cognitive decline, accompanied by slowed movement (bradykinesia), rigid muscles, impaired posture and balance, loss of automatic movements. Majority of PD cases are sporadic, however, some are due to heritable causes, including those caused by mutations in genes encoding α-synuclein, parkin, protein deglycase/Parkinson’s disease protein 7, Pink1, LRRK2, and VPS35 [68].

The *α*-synuclein is the product of *SNCA* gene, and the main component of Lewy bodies (LB) and Lewy neurites (LN). Mutations in *SNCA* (duplications, triplications, or point mutations) might be responsible for certain cases of sporadic PD, or even cause autosomal dominant forms of PD [69]. Interestingly, in LB, LN, and in glial cell inclusions, Hsp90 co-localizes with α-synuclein. Also, Hsp90 level is increased in PD brains, and correlates with increased level of insoluble α-synuclein [70]. In agreement with this study are the results obtained by another group demonstrating the presence of Hsp90 and its co-chaperone, CHP-1, in LB. Interestingly, another co-chaperone, Sgt1, was not found in LB, suggesting distinct roles of these two co-chaperones in the pathogenesis of PD [71]. In yeast, it was found that deletion of Hsp90 (Hsp82) promoted α-synuclein toxicity, specifically by increasing reactive oxygen species (ROS) accumulation [72].

An in vitro study concerning the influence of Hsp90 on α-synuclein demonstrates that in the presence of Hsp90-ATP, α-synuclein oligomers fail to accumulate due to their rapid transformation into fibrils. On the other hand, when ATP is absent, the conversion from the oligomer to fibril state is limited. Thus, authors of this work speculate that inhibition of the Hsp90-ATP cycle by some co-chaperones, such as p23 or STI1/Hop, might promote the formation of soluble oligomers, while stimulation by Aha1 might trigger amyloid fibril formation [73]. A recent study of another research group has revealed that, in vitro, recombinant Hsp90 prevents α-synuclein from aggregating in an ATP-independent manner [12]. Clearly, Hsp90 has an influence on α-synuclein transformation in vitro, however, a number of questions regarding the role of ATP in this process remain to be addressed.

An early study provided evidence that Hsp90 formed a complex with LRRK2 kinase in vivo, and that Hsp90 inhibition disrupted the association with LRRK2, leading to its proteasomal degradation [74]. Another study pointed out that complex formation between Hsp90 and its co-chaperone, Cdc37, plays a crucial role in LRRK2 stabilization. To pursue this further, microglial cell line has been treated with different inhibitors to target the interaction between Hsp90 and Cdc37. Such treatment resulted in disruption of the Hsp90-Cdc37 complex with LRRK2 kinase, consequently leading to destabilization and clearance of LRRK2 [75]. It has been shown that the Hsp90-Cdc37 complex influences the subcellular distribution of its client protein, Pink1 [76]. Another study has indicated that Pink1 level was greatly decreased, reflecting its rapid degradation via the ubiquitin-proteasome pathway, upon treatment with Hsp90 inhibitors, geldanamycin, or novobiocin [77]. A recent study on human fibroblasts suggests that, upon mitochondrial damage, a pathogenic mutated Pink1, p.I368N, has impaired ability to interact with the Hsp90-Cdc37 complex. This mutant is unstable, which results in the loss of mitochondrial quality control [78]. Mutation in the gene encoding Pink1 leads to fatal changes in mitochondrial metabolism, and contributes to selective degeneration of dopaminergic neurons in PD. Until now, the mechanism of this phenomenon remains unclear. Furthermore, it was found that mutations in mitochondrial Pink1 and in the cytosolic parkin, are a cause of autosomally-inherited parkinsonism. Pink1 and parkin together were found to mediate response to mitochondrial stress; they protect the cell from the accumulation of damaged mitochondria [79].

Recently, it was proposed that Hsp90 co-chaperone, p23, contributes to the neurotoxicity in PD. Importantly, p23 knock-down in cultured dopaminergic cells prevented MPP+-mediated neurotoxicity [80].

## 5. Hsp90 in HD

HD is an inherited neurodegenerative disorder, an autosomal dominant disease caused by a CAG trinucleotide repeat expansion in exon 1 of the gene encoding huntingtin (*HTT*). During protein synthesis, these expanded CAG repeats are translated into a series of uninterrupted glutamine residues forming a polyQ tract. PolyQ-expanded HTT is highly prone to aggregation and tends to form inclusion bodies, which are associated with neuronal toxicity/degeneration [81].

It has been reported that Hsp90 interacts with the N-terminus of HTT [13] and together with USP19, modulates aggregation of polyglutamine-expanded ataxin-3 and HTT [82]. Another study provides evidence that there is a physical interaction between wild-type HTT or mutated HTT (mHTT) and Hsp90. Under Hsp90 inhibition, the interaction between these proteins is disrupted, and HTT is cleaved through the ubiquitin-proteasome system [83]. Also, the same study proves that direct inhibition of Hsp90 is crucial for mHTT degradation, and that the effect is due to Hsp90, and not to heat shock response induction and Hsp70 up-regulation. A recent study is in agreement with these data, and indicates that Hsp90 inhibitors may decrease mHTT level in neuroblastoma SH-SY5Y cells [84].

## 6. Hsp90 and Its Co-Chaperones in Prionopathy

Prion diseases are caused by variants of the prion protein which have abnormal shapes. They manifest as different forms of Jakob-Creutzfeldt disease in humans, chronic wasting disease and various forms of scrapie in animals. The human prion protein, PrP, is a small cell-surface glycoprotein. Under physiological conditions, PrP is referred to as PrP^c^, where “^c^” stands for “cellular”. After transformation, PrP becomes highly infectious and is called PrPsc, where “sc” refers to "scrapie" [85]. The physiological role of PrPc is still under investigation. It has been proposed that it can be involved in protection against stress, copper homeostasis, and neuronal excitability. However, these roles of PrPc seem doubtful in the light of recent work, which provides evidence that PrPc is involved in regulation of myelin maintenance, and in processes linked to cellular differentiation, proliferation, adhesion, and control of cell morphology. Also, there is some possible involvement of PrPc in modulation of the circadian rhythm, glucose homeostasis, immune function, and cellular iron uptake [86].

There is extensive experimental evidence of the effect of a Hsp90 co-chaperone, STI1, on PrPc. An early study reported that cellular STI1 associates with recombinant PrP, and both proteins co-immunoprecipitate from HEK 293T cells. Authors used also retinal explants from neonatal rats and mice to induce cell death, and to test a possible protective effect of STI1. They proposed that cooperation between PrPc and STI1 may have a functional influence concerning sensitivity to cell death within the nervous tissue [87]. Another study has suggested that PrPc and STI1 are expressed abundantly, and co-localize in mouse hippocampal neurons. Treatment of primary hippocampal culture with recombinant wild-type STI1 induced neuroprotection, and promoted the process of neuritogenesis [88]. STI1 was also reported to prevent cell death in wild-type astrocytes in a protein kinase A-dependent manner [89]. Subsequently, another study has proposed that STI1 is a potential target of PrPc and that in hippocampal neurons STI1-PrPc cooperation may induce an increase in intracellular Ca2+ level. An inhibitor of the α7nACh receptor, α-bungarotoxin, was able to block PrPc-STI1-mediated signaling, leading to a decrease in neuroprotection and neuritogenesis. Importantly, when the α7nACh receptor was transfected into HEK 293 cells, it formed a functional complex with PrPc and endorsed restoration of the signaling via the PrPc-STI1 complex [90]. Another study on hippocampal neurons focused on the cellular impact behind the interaction between PrPc and STI1. It has been shown that the PrPc-STI1 complex activates protein synthesis, through the PI3K-Akt-mTOR and ERK1/2 pathways. Also, the authors proposed that regulation of protein synthesis is critical for PrPc-STI1 neurotrophic functions. Impairment of this process during PrPsc infection propagation could possibly contribute to neurodegeneration [91].

It is important to consider similarities shared by AD and prion diseases. The co-existence of AD-type pathology in Jakob-Creutzfeldt disease cases was reported early on [92]. PrPC has been shown to co-localize with Aβ in senile plaques [93]. PrPc-Aβ plaques were shown to be present in most Jakob-Creutzfeldt disease patients with associated AD-type pathology [94], and it has been proposed that PrPc may promote Aβ plaque formation [95]. Studies in vitro, utilizing recombinant proteins, pointed out that Hsp90 can modify the conformation of PrPc in the presence of ATP, and it might be required for conformational transition from the physiological PrPc to the infectious PrPsc form [96]. Another study found an age-dependent up-regulation of cortical STI1 in a mouse model of AD, and in the brains of AD-affected individuals. The authors reported that oligomers of Aβ, STI1 and PrP co-existed in a complex. Furthermore, STI1 could efficiently inhibit binding of Aβ oligomers to PrP in vitro, and to cultured mouse primary hippocampal neurons. Treatment with STI1 prevented the Aβ oligomer-induced synaptic loss, neuronal death, and inhibition of long-term potentiation [97].

In yeast, STI1 was shown to influence [PSI+] prion. It was found that cells expressing STI1, and defective in Hsp70 or Hsp90, were cured less efficiently than control cells. The Hsp90 inhibitor, radicicol, abolished curing, supporting the notion that the effect of STI1 is exerted through interaction with Hsp70 and Hsp90 [98]. One in-depth study on a yeast model of prionopathy pointed out the role of Hsp90 and its co-chaperone, an immunophilin homolog, Cpr7 [99]. Authors of this work showed that the yeast homolog of Hsp90, Hsp82, is indirectly involved in prion [URE3] maintenance through interaction with Cpr7. In order to endorse this idea, single knock-out strains of various co-chaperones were prepared. It was found that deletion of Cpr7 affected [URE3] stability. The authors also studied the role of the C-terminus of Hsp82 in [URE3] propagation. They genetically modified this region and the resulting effect was that cells expressing Hsp82 mutant, Hsp82ΔMEEVD, showed [URE3] loss. Importantly, these results were in agreement with a previous study which showed that immunophilins could directly interact with the substrate to assist protein folding. Similarly to Cpr7 in [URE3] propagation, immunophilin FKBP52, present in the human brain, induced aggregation of the pathological tau mutant, tau-P301L [100,101]. Collectively, these data suggest that the role of Cpr7 in [URE3] propagation can be evolutionary conserved in distinct species.

## 7. Concluding Remarks and Therapeutic Perspectives

Aggregated proteins are the main hallmark of different pathologies, including neurodegenerative diseases. Thus, chaperones and co-chaperones, which are responsible for proper protein folding and for preventing protein aggregation, may play a crucial role in protecting cells against accumulation of pathological protein deposits. An essential chaperone and a major regulator of protein folding in all eukaryotic cells is Hsp90. As outlined in this review Hsp90 and its co-chaperones, among them CHIP, Cdc37, FKBP51, FKBP52, p23, PP5, CacyBP/SIP, Aha1, STI1/Hop, Sgt1, CHP-1, and Cpr7, seem to be involved in many cellular processes related to neurodegenerative disorders such as Alzheimer’s disease, Parkinson’s disease, Huntington’s disease and prionopathies (Table 2).

It should be mentioned that Hsp90 inhibitors are studied extensively as potential anticancer drugs, with several compounds being tested at different stages of clinical trials [102,103]. Hsp90 also constitutes a potential drug target in neurological diseases that arise due to accumulation of pathologically folded proteins. The existing data indicate that inhibition of Hsp90 or its co-chaperones, Aha1 and Cdc37, leads to a decrease in formation of tau and Aβ aggregates. Hsp90 inhibition could also lead to degradation of the tau phosphorylating kinases. Of the two cytoplasmic Hsp90 isoforms, Hsp90β is present in all tissues whereas Hsp90α is expressed predominantly in testis and brain [104]. The functions of the two isoforms largely overlap but there is growing evidence that each has unique clients that cannot be chaperoned by the other one [105]. Recently published results indicate that it may be possible to design an Hsp90α-or Hsp90β-specific inhibitor, despite a high degree of similarity between these two isoforms [106,107,108,109]. Moreover, of the two Hsp90 proteins, Hsp90α binds Aha1 and p23, which are co-chaperones that regulate its ATPase activity, more efficiently [110,111]. Thus, applying inhibitors specifically targeting Hsp90α or its interaction with Aha1 might be an efficient way to control tau aggregation with limited side effects [112]. As it is well known, a pathology often associated with Alzheimer’s disease is chronic inflammation [113]. Hsp90 interacts with components of the inflammatory process and Hsp90 inhibitors were shown to counteract inflammation [114,115]. Expression of Hsp90, especially Hsp90α, is elevated at the sites of inflammation suggesting that specific targeting of this isoform may be beneficial in Alzheimer’s disease [116,117]. In conclusion, there are a lot of data collected so far which indicate that Hsp90 may serve as an attractive target for pharmacological intervention in neurodegenerative diseases. If so, future research should concentrate on designing specific, blood–brain barrier penetrating, inhibitors of the Hsp90 isoforms and of their co-chaperones.

## Figures and Tables

**Table 1:** Selected Hsp90 co-chaperones.

Short Name/Gene Name	Full Name
Aha1/*AHSA1*	Activator of Hsp90 ATPase activity 1
CacyBP/SIP/*CACYBP*	Calcyclin Binding Protein/Siah-1 Interacting Protein
Cdc37/*CDC37*	Cell division cycle 37
CHIP/*STUB1*	Carboxyl terminus of Hsp70-interacting protein
CHP-1/*CHORDC1*	Cysteine and histidine rich domain containing protein 1
Cpr7/*CPR7*	Peptidylprolyl isomerase CPR7
FKBP51/FKBP5	FK506 binding protein 5
FKBP52/*FKBP4*	FK506 binding protein 4
PP5/*PP5*	Protein phosphatase 5
p23/*PTGES3*	Co-chaperone p23
Sgt1/*SUGT1*	Suppressor-of-G2-allele-of-skp1
STI1/Hop/*STI1*	Stress-inducible phosphoprotein 1/Hop
USP19/*USP19*	Cytoplasmic Ubiquitin-Specific Protease 19

**Table 2:** Hsp90 co-chaperones and their involvement in processes related to neurodegeneration.

Co-Chaperone	Cellular Processes	Disease
Aha1	Enhances tau aggregation and increases accumulation of insoluble forms; overexpression of Aha1 in a mouse model causes neuronal loss and cognitive impairments [112]	AD
CacyBP/SIP	dephosphorylates tau [49]	AD
Cdc37	Cdc37 co-localizes with tau in neuronal cells; interacts with tau from human brain; knock-down of Cdc37 alters tau phosphorylation [51] Suppression of Cdc37 destabilizes tau and leads to its clearance [53] Responsible for LRRK2 and Pink1 stabilization [75,77] Influences subcellular distribution of Pink1 [76]	AD, PD
CHIP	Promotes tau ubiquitination [53]	AD
CHP-1	Present in LB of PD [71]	PD
Cpr7	Involved in yeast prion [URE3] maintenance [99]	Prionopathies
FKBP51	Up-regulated in aged and AD brain [56] Influences tau stabilization [57,58] Knock-down of FKBP51causes decrease in total tau level; overexpression of FKBP5 causes an increase in phosphorylated and total tau level [57]	AD
FKBP52	Low level in AD brains [59] Overexpression of FKBP52 has no effect on phosphorylation and total tau level [57] Detected together with tau in the autophagy-endolysosomal system; decreased level correlates with NFT formation [59] Induces aggregation of the pathological tau mutant, tau-P301L [100,101]	AD
PP5	Dephosphorylates tau [48,50] Has lower activity in AD brain [48]	AD
p23	Depletion of p23 causes significant decrease in total and phosphorylated tau level [42] Contributes to neurotoxicity in PD [80]	AD, PD
Sgt1	Absent in LB of PD brain [71] Decreased level in AD brain [55]	PD, AD
STI1/Hop	Influences clearance of tau [54] Co-immunoprecipitates with PrP [87] PrPc-STI1 influences sensitivity to cell death within the nervous tissue [87] Promotes neuroprotection and neuritogenesis [88,90,91] PrPc-STI1 activates protein synthesis through the PI3K–Akt–mTOR and ERK1/2 pathways [91] Prevents formation of the Aβ oligomers [97] Mediates curative effect on [*PSI+*] prion [98]	AD, prionopathies
USP19	Increases HTT level, promotes aggregation of the polyQ-expanded HTT [82]	HT

## Abbreviations

Aβ	amyloid β
α7nAChR	α7 nicotinic acetylcholine receptor
α-bungarotoxin	inhibitor of α7nAChR
AD	Alzheimer’s disease
AKT	protein kinase B/serine/threonine kinase 1
ALS	amyotrophic lateral sclerosis
APP	amyloid precursor protein
ATP	adenosine triphosphate
Cdk5	cyclin-dependent kinase 5
IL-6	interleukin 6
ERK1/2	mitogen-activated protein kinase 1 and 2
FKBP51	FK506 binding protein 5
FKBP52	FK506-binding protein 4
HD	Huntington’s disease
HSE	heat-shock-factor element
HSF1	heat shock factor 1
HTT	huntingtin
LB	Lewy bodies
LN	Lewy neurites
LRRK2	leucine-rich repeat kinase 2
MPP+	1-methyl-4-phenyl pyridinium
mTOR	mechanistic target of rapamycin kinase
NFTs	neurofibrillary tangles
Parkin	E3 ubiquitin ligase
PD	Parkinson’s disease
PI3K	phosphatidylinositol kinase
Pink	PTEN-induced kinase 1
PrP	prion protein
PTMs	posttranslational modifications
SNCA	α-synuclein gene
TSEs	transmissible spongiform encephalopathies
TLR4	toll-like receptor 4
TNFα	tumor necrosis factor alpha
USP19	ubiquitin-specific protease 19
VPS35	vacuolar protein sorting 35

## References

[1] Ciechanover A., Kwon Y.T. (2015). Degradation of misfolded proteins in neurodegenerative diseases: Therapeutic targets and strategies. Exp. Mol. Med..

[2] Cuanalo-Contreras K., Mukherjee A., Soto C. (2013). Role of protein misfolding and proteostasis deficiency in protein misfolding diseases and aging. Int. J. Cell Biol..

[3] Pace M.C., Xu G., Fromholt S., Howard J., Crosby K., Giasson B.I., Lewis J., Borchelt D.R. (2018). Changes in proteome solubility indicate widespread proteostatic disruption in mouse models of neurodegenerative disease. Acta. Neuropathol..

[4] Garrido C., Gurbuxani S., Ravagnan L., Kroemer G. (2001). Heat shock proteins: Endogenous modulators of apoptotic cell death. Biochem. Biophys. Res. Commun..

[5] Castro J.P., Wardelmann K., Grune T., Kleinridders A. (2018). Mitochondrial Chaperones in the Brain: Safeguarding Brain Health and Metabolism?. Front Endocrinol. (Lausanne).

[6] Jackson S.E. (2013). Hsp90: Structure and function. Top Curr. Chem..

[7] Luo W., Sun W., Taldone T., Rodina A., Chiosis G. (2010). Heat shock protein 90 in neurodegenerative diseases. Mol. Neurodegener..

[8] Picard D. (2002). Heat-shock protein 90, a chaperone for folding and regulation. Cell Mol. Life Sci..

[9] Whitesell L., Lindquist S.L. (2005). HSP90 and the chaperoning of cancer. Nat. Rev. Cancer.

[10] Taipale M., Jarosz D.F., Lindquist S. (2010). HSP90 at the hub of protein homeostasis: Emerging mechanistic insights. Nat. Rev. Mol. Cell Biol..

[11] Shelton L.B., Koren J., Blair L.J. (2017). Imbalances in the Hsp90 Chaperone Machinery: Implications for Tauopathies. Front Neurosci..

[12] Daturpalli S., Waudby C.A., Meehan S., Jackson S.E. (2013). Hsp90 inhibits alpha-synuclein aggregation by interacting with soluble oligomers. J. Mol. Biol..

[13] He W.T., Xue W., Gao Y.G., Hong J.Y., Yue H.W., Jiang L.L., Hu H.Y. (2017). HSP90 recognizes the N-terminus of huntingtin involved in regulation of huntingtin aggregation by USP19. Sci. Rep..

[14] Hahn J.S. (2009). The Hsp90 chaperone machinery: From structure to drug development. BMB Rep..

[15] Sreedhar A.S., Kalmar E., Csermely P., Shen Y.F. (2004). Hsp90 isoforms: Functions, expression and clinical importance. FEBS Lett..

[16] Obermann W.M., Sondermann H., Russo A.A., Pavletich N.P., Hartl F.U. (1998). In vivo function of Hsp90 is dependent on ATP binding and ATP hydrolysis. J. Cell Biol..

[17] Panaretou B., Prodromou C., Roe S.M., O’Brien R., Ladbury J.E., Piper P.W., Pearl L.H. (1998). ATP binding and hydrolysis are essential to the function of the Hsp90 molecular chaperone in vivo. EMBO J..

[18] Schopf F.H., Biebl M.M., Buchner J. (2017). The HSP90 chaperone machinery. Nat. Rev. Mol. Cell Biol..

[19] Mollapour M., Neckers L. (2012). Post-translational modifications of Hsp90 and their contributions to chaperone regulation. Biochim. Biophys Acta..

[20] Mayer M.P., Le Breton L. (2015). Hsp90: Breaking the symmetry. Mol. Cell.

[21] Prodromou C. (2016). Mechanisms of Hsp90 regulation. Biochem. J..

[22] Sima S., Richter K. (2018). Regulation of the Hsp90 system. Biochim. Biophys Acta. Mol. Cell Res..

[23] Akerfelt M., Morimoto R.I., Sistonen L. (2010). Heat shock factors: Integrators of cell stress, development and lifespan. Nat. Rev. Mol. Cell Biol..

[24] Prodromou C. (2017). Regulatory Mechanisms of Hsp90. Biochem. Mol. Biol. J..

[25] Retzlaff M., Stahl M., Eberl H.C., Lagleder S., Beck J., Kessler H., Buchner J. (2009). Hsp90 is regulated by a switch point in the C-terminal domain. EMBO Rep..

[26] Soroka J., Wandinger S.K., Mausbacher N., Schreiber T., Richter K., Daub H., Buchner J. (2012). Conformational switching of the molecular chaperone Hsp90 via regulated phosphorylation. Mol. Cell.

[27] Wandinger S.K., Suhre M.H., Wegele H., Buchner J. (2006). The phosphatase Ppt1 is a dedicated regulator of the molecular chaperone Hsp90. EMBO J..

[28] Nguyen M.T.N., Kniess R.A., Daturpalli S., Le Breton L., Ke X., Chen X., Mayer M.P. (2017). Isoform-Specific Phosphorylation in Human Hsp90beta Affects Interaction with Clients and the Cochaperone Cdc37. J. Mol. Biol..

[29] Scroggins B.T., Robzyk K., Wang D., Marcu M.G., Tsutsumi S., Beebe K., Cotter R.J., Felts S., Toft D., Karnitz L. (2007). An acetylation site in the middle domain of Hsp90 regulates chaperone function. Mol Cell.

[30] Melis V., Zabke C., Stamer K., Magbagbeolu M., Schwab K., Marschall P., Veh R.W., Bachmann S., Deiana S., Moreau P.H. (2015). Different pathways of molecular pathophysiology underlie cognitive and motor tauopathy phenotypes in transgenic models for Alzheimer’s disease and frontotemporal lobar degeneration. Cell Mol. Life Sci..

[31] Selkoe D.J., Schenk D. (2003). Alzheimer’s disease: Molecular understanding predicts amyloid-based therapeutics. Annu Rev. Pharmacol. Toxicol..

[32] Esch F.S., Keim P.S., Beattie E.C., Blacher R.W., Culwell A.R., Oltersdorf T., McClure D., Ward P.J. (1990). Cleavage of amyloid beta peptide during constitutive processing of its precursor. Science.

[33] Paul S., Mahanta S. (2014). Association of heat-shock proteins in various neurodegenerative disorders: Is it a master key to open the therapeutic door?. Mol. Cell Biochem..

[34] Kadavath H., Hofele R.V., Biernat J., Kumar S., Tepper K., Urlaub H., Mandelkow E., Zweckstetter M. (2015). Tau stabilizes microtubules by binding at the interface between tubulin heterodimers. Proc. Natl. Acad. Sci USA.

[35] Baas P.W., Pienkowski T.P., Cimbalnik K.A., Toyama K., Bakalis S., Ahmad F.J., Kosik K.S. (1994). Tau confers drug stability but not cold stability to microtubules in living cells. J. Cell Sci..

[36] Lee G., Rook S.L. (1992). Expression of tau protein in non-neuronal cells: Microtubule binding and stabilization. J. Cell Sci..

[37] Qiang L., Sun X., Austin T.O., Muralidharan H., Jean D.C., Liu M., Yu W., Baas P.W. (2018). Tau Does Not Stabilize Axonal Microtubules but Rather Enables Them to Have Long Labile Domains. Curr. Biol..

[38] Tanemura K., Murayama M., Akagi T., Hashikawa T., Tominaga T., Ichikawa M., Yamaguchi H., Takashima A. (2002). Neurodegeneration with tau accumulation in a transgenic mouse expressing V337M human tau. J. Neurosci..

[39] Rodriguez-Callejas J.D., Fuchs E., Perez-Cruz C. (2016). Evidence of Tau Hyperphosphorylation and Dystrophic Microglia in the Common Marmoset. Front. Aging Neurosci..

[40] Alonso A.D., Cohen L.S., Corbo C., Morozova V., ElIdrissi A., Phillips G., Kleiman F.E. (2018). Hyperphosphorylation of Tau Associates With Changes in Its Function Beyond Microtubule Stability. Front. Cell Neurosci..

[41] Salminen A., Ojala J., Kaarniranta K., Hiltunen M., Soininen H. (2011). Hsp90 regulates tau pathology through co-chaperone complexes in Alzheimer’s disease. Prog. Neurobiol..

[42] Dickey C.A., Kamal A., Lundgren K., Klosak N., Bailey R.M., Dunmore J., Ash P., Shoraka S., Zlatkovic J., Eckman C.B. (2007). The high-affinity HSP90-CHIP complex recognizes and selectively degrades phosphorylated tau client proteins. J. Clin. Invest..

[43] Basso A.D., Solit D.B., Chiosis G., Giri B., Tsichlis P., Rosen N. (2002). Akt forms an intracellular complex with heat shock protein 90 (Hsp90) and Cdc37 and is destabilized by inhibitors of Hsp90 function. J. Biol. Chem..

[44] Miyata Y., Nakamoto H., Neckers L. (2013). The therapeutic target Hsp90 and cancer hallmarks. Curr. Pharm. Des..

[45] Cruz J.C., Tseng H.C., Goldman J.A., Shih H., Tsai L.H. (2003). Aberrant Cdk5 activation by p25 triggers pathological events leading to neurodegeneration and neurofibrillary tangles. Neuron.

[46] Lee V.M., Brunden K.R., Hutton M., Trojanowski J.Q. (2011). Developing therapeutic approaches to tau, selected kinases, and related neuronal protein targets. Cold Spring Harb Perspect Med..

[47] Luo W., Dou F., Rodina A., Chip S., Kim J., Zhao Q., Moulick K., Aguirre J., Wu N., Greengard P. (2007). Roles of heat-shock protein 90 in maintaining and facilitating the neurodegenerative phenotype in tauopathies. Proc. Natl. Acad. Sci. USA.

[48] Liu F., Grundke-Iqbal I., Iqbal K., Gong C.X. (2005). Contributions of protein phosphatases PP1, PP2A, PP2B and PP5 to the regulation of tau phosphorylation. Eur. J. Neurosci..

[49] Wasik U., Schneider G., Mietelska-Porowska A., Mazurkiewicz M., Fabczak H., Weis S., Zabke C., Harrington C.R., Filipek A., Niewiadomska G. (2013). Calcyclin binding protein and Siah-1 interacting protein in Alzheimer’s disease pathology: Neuronal localization and possible function. Neurobiol. Aging.

[50] Liu F., Iqbal K., Grundke-Iqbal I., Rossie S., Gong C.X. (2005). Dephosphorylation of tau by protein phosphatase 5: Impairment in Alzheimer’s disease. J. Biol. Chem..

[51] Jinwal U.K., Trotter J.H., Abisambra J.F., Koren J., Lawson L.Y., Vestal G.D., O’Leary J.C., Johnson A.G., Jin Y., Jones J.R. (2011). The Hsp90 kinase co-chaperone Cdc37 regulates tau stability and phosphorylation dynamics. J. Biol. Chem..

[52] Mukrasch M.D., Bibow S., Korukottu J., Jeganathan S., Biernat J., Griesinger C., Mandelkow E., Zweckstetter M. (2009). Structural polymorphism of 441-residue tau at single residue resolution. PLoS Biol..

[53] Dickey C.A., Koren J., Zhang Y.J., Xu Y.F., Jinwal U.K., Birnbaum M.J., Monks B., Sun M., Cheng J.Q., Patterson C. (2008). Akt and CHIP coregulate tau degradation through coordinated interactions. Proc. Natl. Acad. Sci. USA.

[54] Ambegaokar S.S., Jackson G.R. (2011). Functional genomic screen and network analysis reveal novel modifiers of tauopathy dissociated from tau phosphorylation. Hum. Mol. Genet..

[55] Spiechowicz M., Bernstein H.G., Dobrowolny H., Lesniak W., Mawrin C., Bogerts B., Kuznicki J., Filipek A. (2006). Density of Sgt1-immunopositive neurons is decreased in the cerebral cortex of Alzheimer’s disease brain. Neurochem. Int..

[56] Blair L.J., Nordhues B.A., Hill S.E., Scaglione K.M., O’Leary J.C., Fontaine S.N., Breydo L., Zhang B., Li P., Wang L. (2013). Accelerated neurodegeneration through chaperone-mediated oligomerization of tau. J. Clin. Invest..

[57] Jinwal U.K., Koren J., Borysov S.I., Schmid A.B., Abisambra J.F., Blair L.J., Johnson A.G., Jones J.R., Shults C.L., O’Leary J.C. (2010). The Hsp90 cochaperone, FKBP51, increases Tau stability and polymerizes microtubules. J. Neurosci..

[58] Oroz J., Chang B.J., Wysoczanski P., Lee C.T., Perez-Lara A., Chakraborty P., Hofele R.V., Baker J.D., Blair L.J., Biernat J. (2018). Structure and pro-toxic mechanism of the human Hsp90/PPIase/Tau complex. Nat. Commun..

[59] Meduri G., Guillemeau K., Dounane O., Sazdovitch V., Duyckaerts C., Chambraud B., Baulieu E.E., Giustiniani J. (2016). Caspase-cleaved Tau-D(421) is colocalized with the immunophilin FKBP52 in the autophagy-endolysosomal system of Alzheimer’s disease neurons. Neurobiol. Aging.

[60] LeMaster D.M., Hernandez G. (2015). Conformational Dynamics in FKBP Domains: Relevance to Molecular Signaling and Drug Design. Curr. Mol. Pharmacol..

[61] Reese L.C., Zhang W., Dineley K.T., Kayed R., Taglialatela G. (2008). Selective induction of calcineurin activity and signaling by oligomeric amyloid beta. Aging Cell.

[62] Dineley K.T., Hogan D., Zhang W.R., Taglialatela G. (2007). Acute inhibition of calcineurin restores associative learning and memory in Tg2576 APP transgenic mice. Neurobiol. Learn Mem..

[63] Dineley K.T., Kayed R., Neugebauer V., Fu Y., Zhang W., Reese L.C., Taglialatela G. (2010). Amyloid-beta oligomers impair fear conditioned memory in a calcineurin-dependent fashion in mice. J. Neurosci. Res..

[64] Taglialatela G., Rastellini C., Cicalese L. (2015). Reduced Incidence of Dementia in Solid Organ Transplant Patients Treated with Calcineurin Inhibitors. J. Alzheimers Dis..

[65] Kakimura J., Kitamura Y., Takata K., Umeki M., Suzuki S., Shibagaki K., Taniguchi T., Nomura Y., Gebicke-Haerter P.J., Smith M.A. (2002). Microglial activation and amyloid-beta clearance induced by exogenous heat-shock proteins. FASEB J..

[66] Wang W.Y., Tan M.S., Yu J.T., Tan L. (2015). Role of pro-inflammatory cytokines released from microglia in Alzheimer’s disease. Ann. Transl. Med..

[67] Evans C.G., Wisen S., Gestwicki J.E. (2006). Heat shock proteins 70 and 90 inhibit early stages of amyloid beta-(1-42) aggregation in vitro. J. Biol. Chem..

[68] Hernandez D.G., Reed X., Singleton A.B. (2016). Genetics in Parkinson disease: Mendelian versus non-Mendelian inheritance. J. Neurochem..

[69] Tan E.K., Chandran V.R., Fook-Chong S., Shen H., Yew K., Teoh M.L., Yuen Y., Zhao Y. (2005). Alpha-synuclein mRNA expression in sporadic Parkinson’s disease. Mov. Disord..

[70] Uryu K., Richter-Landsberg C., Welch W., Sun E., Goldbaum O., Norris E.H., Pham C.T., Yazawa I., Hilburger K., Micsenyi M. (2006). Convergence of heat shock protein 90 with ubiquitin in filamentous alpha-synuclein inclusions of alpha-synucleinopathies. Am. J. Pathol..

[71] Bohush A., Niewiadomska G., Weis S., Filipek A. (2019). HSP90 and Its Novel Co-Chaperones, SGT1 and CHP-1, in Brain of Patients with Parkinson’s Disease and Dementia with Lewy Bodies. J. Parkinsons Dis..

[72] Liang J., Clark-Dixon C., Wang S., Flower T.R., Williams-Hart T., Zweig R., Robinson L.C., Tatchell K., Witt S.N. (2008). Novel suppressors of alpha-synuclein toxicity identified using yeast. Hum. Mol. Genet..

[73] Falsone S.F., Kungl A.J., Rek A., Cappai R., Zangger K. (2009). The molecular chaperone Hsp90 modulates intermediate steps of amyloid assembly of the Parkinson-related protein alpha-synuclein. J. Biol. Chem..

[74] Wang L., Xie C., Greggio E., Parisiadou L., Shim H., Sun L., Chandran J., Lin X., Lai C., Yang W.J. (2008). The chaperone activity of heat shock protein 90 is critical for maintaining the stability of leucine-rich repeat kinase 2. J. Neurosci..

[75] Narayan M., Zhang J., Braswell K., Gibson C., Zitnyar A., Lee D.C., Varghese-Gupta S., Jinwal U.K. (2015). Withaferin A Regulates LRRK2 Levels by Interfering with the Hsp90- Cdc37 Chaperone Complex. Curr. Aging Sci..

[76] Weihofen A., Ostaszewski B., Minami Y., Selkoe D.J. (2008). Pink1 Parkinson mutations, the Cdc37/Hsp90 chaperones and Parkin all influence the maturation or subcellular distribution of Pink1. Hum. Mol. Genet..

[77] Moriwaki Y., Kim Y.J., Ido Y., Misawa H., Kawashima K., Endo S., Takahashi R. (2008). L347P PINK1 mutant that fails to bind to Hsp90/Cdc37 chaperones is rapidly degraded in a proteasome-dependent manner. Neurosci. Res..

[78] Ando M., Fiesel F.C., Hudec R., Caulfield T.R., Ogaki K., Gorka-Skoczylas P., Koziorowski D., Friedman A., Chen L., Dawson V.L. (2017). The PINK1 p.I368N mutation affects protein stability and ubiquitin kinase activity. Mol. Neurodegener..

[79] Scarffe L.A., Stevens D.A., Dawson V.L., Dawson T.M. (2014). Parkin and PINK1: Much more than mitophagy. Trends Neurosci..

[80] Rane A., Rajagopalan S., Ahuja M., Thomas B., Chinta S.J., Andersen J.K. (2018). Hsp90 Co-chaperone p23 contributes to dopaminergic mitochondrial stress via stabilization of PHD2: Implications for Parkinson’s disease. Neurotoxicology.

[81] Gusella J.F., MacDonald M.E. (2006). Huntington’s disease: Seeing the pathogenic process through a genetic lens. Trends Biochem Sci.

[82] He W.T., Zheng X.M., Zhang Y.H., Gao Y.G., Song A.X., van der Goot F.G., Hu H.Y. (2016). Cytoplasmic Ubiquitin-Specific Protease 19 (USP19) Modulates Aggregation of Polyglutamine-Expanded Ataxin-3 and Huntingtin through the HSP90 Chaperone. PLoS ONE.

[83] Baldo B., Weiss A., Parker C.N., Bibel M., Paganetti P., Kaupmann K. (2012). A screen for enhancers of clearance identifies huntingtin as a heat shock protein 90 (Hsp90) client protein. J. Biol. Chem..

[84] Orozco-Diaz R., Sanchez-Alvarez A., Hernandez-Hernandez J.M., Tapia-Ramirez J. (2019). The interaction between RE1-silencing transcription factor (REST) and heat shock protein 90 as new therapeutic target against Huntington’s disease. PLoS ONE.

[85] Wille H., Requena J.R. (2018). The Structure of PrP(Sc) Prions. Pathogens.

[86] Castle A.R., Gill A.C. (2017). Physiological Functions of the Cellular Prion Protein. Front Mol Biosci.

[87] Zanata S.M., Lopes M.H., Mercadante A.F., Hajj G.N., Chiarini L.B., Nomizo R., Freitas A.R., Cabral A.L., Lee K.S., Juliano M.A. (2002). Stress-inducible protein 1 is a cell surface ligand for cellular prion that triggers neuroprotection. EMBO J..

[88] Lopes M.H., Hajj G.N., Muras A.G., Mancini G.L., Castro R.M., Ribeiro K.C., Brentani R.R., Linden R., Martins V.R. (2005). Interaction of cellular prion and stress-inducible protein 1 promotes neuritogenesis and neuroprotection by distinct signaling pathways. J. Neurosci..

[89] Arantes C., Nomizo R., Lopes M.H., Hajj G.N., Lima F.R., Martins V.R. (2009). Prion protein and its ligand stress inducible protein 1 regulate astrocyte development. Glia.

[90] Beraldo F.H., Arantes C.P., Santos T.G., Queiroz N.G., Young K., Rylett R.J., Markus R.P., Prado M.A., Martins V.R. (2010). Role of alpha7 nicotinic acetylcholine receptor in calcium signaling induced by prion protein interaction with stress-inducible protein 1. J. Biol. Chem..

[91] Roffe M., Beraldo F.H., Bester R., Nunziante M., Bach C., Mancini G., Gilch S., Vorberg I., Castilho B.A., Martins V.R. (2010). Prion protein interaction with stress-inducible protein 1 enhances neuronal protein synthesis via mTOR. Proc. Natl. Acad. Sci. USA.

[92] Hainfellner J.A., Wanschitz J., Jellinger K., Liberski P.P., Gullotta F., Budka H. (1998). Coexistence of Alzheimer-type neuropathology in Creutzfeldt-Jakob disease. Acta. Neuropathol..

[93] Voigtlander T., Kloppel S., Birner P., Jarius C., Flicker H., Verghese-Nikolakaki S., Sklaviadis T., Guentchev M., Budka H. (2001). Marked increase of neuronal prion protein immunoreactivity in Alzheimer’s disease and human prion diseases. Acta. Neuropathol..

[94] Del Bo R., Scarlato M., Ghezzi S., Martinelli-Boneschi F., Fenoglio C., Galimberti G., Galbiati S., Virgilio R., Galimberti D., Ferrarese C. (2006). Is M129V of PRNP gene associated with Alzheimer’s disease? A case-control study and a meta-analysis. Neurobiol. Aging.

[95] Schwarze-Eicker K., Keyvani K., Gortz N., Westaway D., Sachser N., Paulus W. (2005). Prion protein (PrPc) promotes beta-amyloid plaque formation. Neurobiol. Aging.

[96] Sakasegawa Y., Hachiya N.S., Kaneko K., Kitamoto T. (2005). Hsp90 modifies the conformation of recombinant mouse prion protein in vitro. Prions.

[97] Maciejewski A., Ostapchenko V.G., Beraldo F.H., Prado V.F., Prado M.A., Choy W.Y. (2016). Domains of STIP1 responsible for regulating PrPC-dependent amyloid-beta oligomer toxicity. Biochem. J..

[98] Reidy M., Masison D.C. (2010). Sti1 regulation of Hsp70 and Hsp90 is critical for curing of Saccharomyces cerevisiae [PSI+] prions by Hsp104. Mol. Cell Biol..

[99] Kumar N., Gaur D., Gupta A., Puri A., Sharma D. (2015). Hsp90-Associated Immunophilin Homolog Cpr7 Is Required for the Mitotic Stability of [URE3] Prion in Saccharomyces cerevisiae. PLoS Genet..

[100] Giustiniani J., Chambraud B., Sardin E., Dounane O., Guillemeau K., Nakatani H., Paquet D., Kamah A., Landrieu I., Lippens G. (2014). Immunophilin FKBP52 induces Tau-P301L filamentous assembly in vitro and modulates its activity in a model of tauopathy. Proc. Natl. Acad. Sci. USA.

[101] Giustiniani J., Guillemeau K., Dounane O., Sardin E., Huvent I., Schmitt A., Hamdane M., Buee L., Landrieu I., Lippens G. (2015). The FK506-binding protein FKBP52 in vitro induces aggregation of truncated Tau forms with prion-like behavior. FASEB J..

[102] Yuno A., Lee M.J., Lee S., Tomita Y., Rekhtman D., Moore B., Trepel J.B. (2018). Clinical Evaluation and Biomarker Profiling of Hsp90 Inhibitors. Methods Mol. Biol..

[103] Jhaveri K., Taldone T., Modi S., Chiosis G. (2012). Advances in the clinical development of heat shock protein 90 (Hsp90) inhibitors in cancers. Biochim. Biophys Acta..

[104] Wu Y., Zheng X., Ding Y., Zhou M., Wei Z., Liu T., Liao K. (2019). The molecular chaperone Hsp90alpha deficiency causes retinal degeneration by disrupting Golgi organization and vesicle transportation in photoreceptors. J. Mol. Cell Biol..

[105] Zuehlke A.D., Beebe K., Neckers L., Prince T. (2015). Regulation and function of the human HSP90AA1 gene. Gene.

[106] Mahanta S., Pilla S., Paul S. (2013). Design of novel Geldanamycin analogue hsp90 alpha-inhibitor in silico for breast cancer therapy. Med. Hypotheses.

[107] Gupta U.K., Mahanta S., Paul S. (2013). In silico design of small peptide-based Hsp90 inhibitor: A novel anticancer agent. Med. Hypotheses.

[108] Yim K.H., Prince T.L., Qu S., Bai F., Jennings P.A., Onuchic J.N., Theodorakis E.A., Neckers L. (2016). Gambogic acid identifies an isoform-specific druggable pocket in the middle domain of Hsp90beta. Proc. Natl. Acad. Sci. USA.

[109] Stiegler S.C., Rubbelke M., Korotkov V.S., Weiwad M., John C., Fischer G., Sieber S.A., Sattler M., Buchner J. (2017). A chemical compound inhibiting the Aha1-Hsp90 chaperone complex. J. Biol. Chem..

[110] Synoradzki K., Bieganowski P. (2015). Middle domain of human Hsp90 isoforms differentially binds Aha1 in human cells and alters Hsp90 activity in yeast. Biochim. Biophys Acta..

[111] Synoradzki K., Miszta P., Kazlauskas E., Mickeviciute A., Michailoviene V., Matulis D., Filipek S., Bieganowski P. (2018). Interaction of the middle domains stabilizes Hsp90alpha dimer in a closed conformation with high affinity for p23. Biol. Chem..

[112] Shelton L.B., Baker J.D., Zheng D., Sullivan L.E., Solanki P.K., Webster J.M., Sun Z., Sabbagh J.J., Nordhues B.A., Koren J. (2017). Hsp90 activator Aha1 drives production of pathological tau aggregates. Proc. Natl. Acad. Sci. USA.

[113] McGeer P.L., Rogers J., McGeer E.G. (2016). Inflammation, Antiinflammatory Agents, and Alzheimer’s Disease: The Last 22 Years. J. Alzheimers Dis..

[114] Li F., Song X., Su G., Wang Y., Wang Z., Qing S., Jia J., Huang L., Zheng K. (2019). AT-533, a Hsp90 inhibitor, attenuates HSV-1-induced inflammation. Biochem. Pharmacol..

[115] Mu H., Wang L., Zhao L. (2017). HSP90 inhibition suppresses inflammatory response and reduces carotid atherosclerotic plaque formation in ApoE mice. Cardiovasc Ther.

[116] Kakeda M., Arock M., Schlapbach C., Yawalkar N. (2014). Increased expression of heat shock protein 90 in keratinocytes and mast cells in patients with psoriasis. J. Am. Acad. Dermatol..

[117] Metz K., Ezernieks J., Sebald W., Duschl A. (1996). Interleukin-4 upregulates the heat shock protein Hsp90alpha and enhances transcription of a reporter gene coupled to a single heat shock element. FEBS Lett..

